# Robust Bound States in the Continuum in Mirror‐Symmetric Terahertz Metasurfaces

**DOI:** 10.1002/nap2.70221

**Published:** 2026-07-17

**Authors:** Die Zou, Zhen Zhou, Xia Cao, Rui Zhu, Keyu Tan, Zhiyong Wang, Xiaohua Xing, Jianquan Yao, Jianing Chen, Liang Wu

**Affiliations:** ^1^ Key Laboratory of Optoelectronics Information and Technology (Ministry of Education), College of Precision Instrument and Optoelectronics Engineering Tianjin University Tianjin China; ^2^ Key Laboratory of Modern Engineering Mechanics (Ministry of Education) College of Mechanical Engineering Tianjin University Tianjin China; ^3^ The Institute of Physics, Chinese Academy of Sciences & School of Physical Sciences University of Chinese Academy of Sciences Beijing China

**Keywords:** bound states in the continuum, in plane mirror symmetry, symmetry breaking, terahertz photonics

## Abstract

Conventional *C*_2_‐symmetric metasurfaces supporting symmetry‐protected bound states in the continuum (SP‐BICs) are often hindered by sensitivity to fabrication errors. Here, we demonstrate that for metasurfaces composed of resonant dimers possessing local inversion symmetry, the SP‐BIC mode remains robustly sustained at the *Γ*‐point as long as in‐plane mirror symmetry is preserved, even if the dimers undergo spatial displacement within the lattice that breaks global periodic inversion symmetry. This characteristic endows the design with a unique offset‐robustness, significantly relaxing the stringent requirements for fabrication precision. Build on this, we introduce an asymmetry parameter (*α*) to break the mirror symmetry (and consequently the local inversion symmetry), enabling the transformation of the BIC into a quasi‐BIC (QBIC). Experimental and simulation results show that the radiative loss of the QBIC can be tuned via *α*, with the quality factor following a power‐law scaling of Q ∝ *α* ^−1.95^. Momentum‐space and near‐field analyses indicate that this transition is accompanied by the splitting of topological singularities and the breakdown of multipole interference equilibrium. This study demonstrates that local inversion symmetry is the physical origin of BIC formation, whereas mirror symmetry ensures the critical tolerance to displacement, providing a theoretical basis and design approach for high‐performance, process‐tolerant terahertz photonic devices.

## Graphical abstract

An ideal BIC, supported by local inversion symmetry in a mirror‐symmetric metasurface, transforms into a QBIC by introducing an asymmetry parameter *α*. This transition yields a finite Q‐factor following Q ∝ *α* ^−1.95^, whereas the singular polarization state splits into two distinct circularly polarized points. This framework ensures robust BIC formation at the *Γ*‐point, maintaining high performance even under spatial lattice displacements.

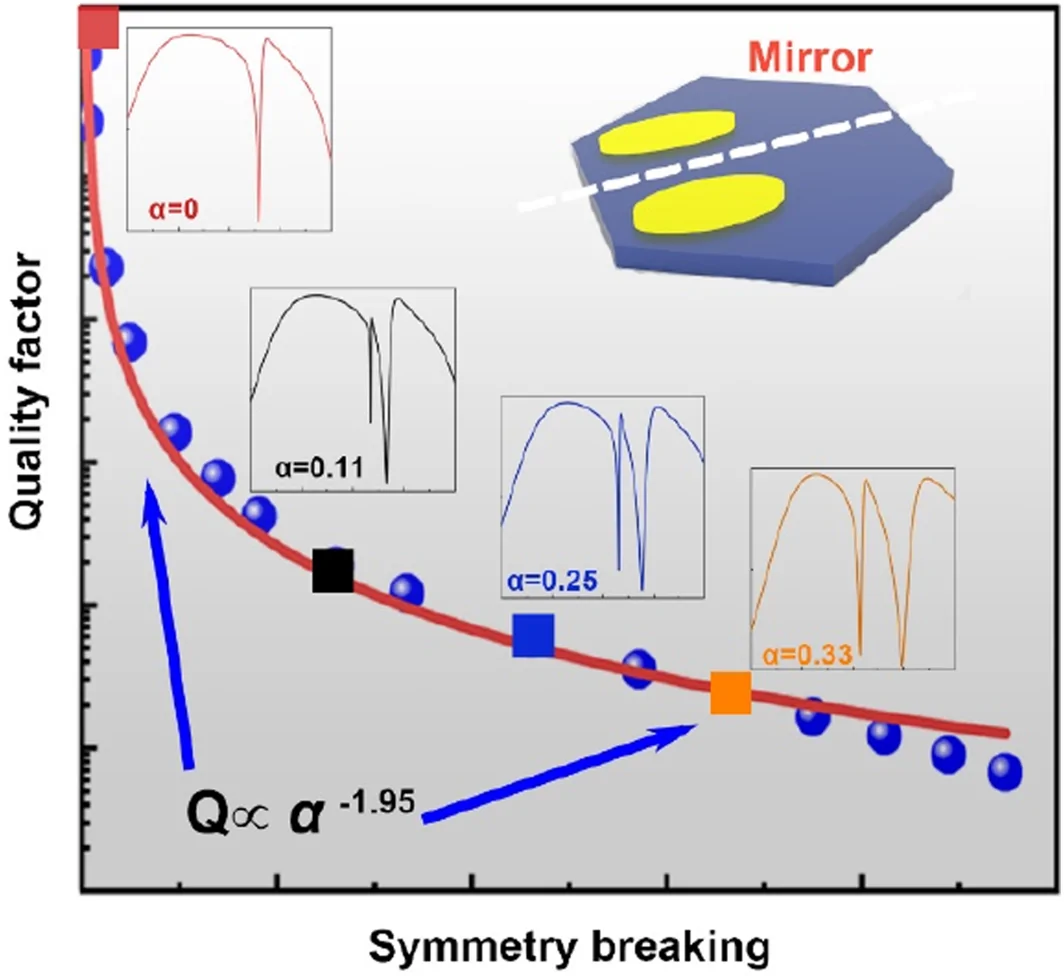

## 1 Introduction

Ideal BICs possess infinitely high quality factors (Q‐factors) and strong field localization capabilities [1, 2, 3, 4, 5]. However, their nonradiative nature also prevents coupling with free space [6, 7], limiting their practical applications in sensing, lasing, nonlinear optics, and other fields. To address this contradiction, the main strategy is to transform ideal BICs into QBICs with finite Q‐factors by introducing controlled structural perturbations [8, 9, 10, 11, 12]. Here, SP‐BICs based on specific spatial symmetries (such as rotational symmetry) have attracted widespread attention due to their high design flexibility [6, 13, 14, 15, 16]. Classical theory establishes that in‐plane inversion symmetry is essential for realizing an ideal SP‐BIC, as it prohibits radiative coupling by matching mode parity with the radiation field [17, 18, 19, 20]. Currently, the majority of research focuses on structures with global inversion symmetry [21, 22, 23, 24]. However, such designs typically require the resonant units to be precisely centered within the lattice. Any displacement of the unit center caused by fabrication errors directly breaks the global symmetry, leading to a sharp deterioration in BIC performance and severely limiting the fabrication tolerance of practical devices [25, 26].

In contrast, the approach of realizing SP‐BIC based on in‐plane mirror symmetry offers notable advantages in terms of practical applicability. Compared to in‐plane inversion symmetry, a single mirror symmetry only requires the structure to exhibit reflection symmetry across a certain plane (i.e., coordinates satisfying (*x*, *y*) = (*x*, −*y*) or (*x*, *y*) = (−*x*, *y*)). This single‐dimensional symmetry constraint relaxes fabrication precision requirements and improves tolerance to process variations such as spatial offsets, ensuring robust performance despite manufacturing imperfections [17, 27]. Consequently, it aligns more closely with realistic fabrication conditions and device application requirements. Moreover, in schemes that achieve BICs through Brillouin zone folding, the structures often inherently retain some degree of mirror symmetry [28, 29, 30]. Nevertheless, how to synergistically exploit mirror symmetry and local inversion symmetry, and to systematically elucidate the underlying physical mechanisms and fabrication advantages within a unified theoretical framework, remains to be thoroughly investigated.

Here, we propose a mirror‐symmetry‐based metasurface that successfully decouples BIC formation from the requirement of precise global alignment of the resonant unit. In this design, the local inversion symmetry of individual dimers governs BIC formation and the cancellation of far‐field radiation, whereas the in‐plane mirror symmetry maintained by the overall lattice provides geometric protection, ensuring that the BIC persists even when the dimer undergoes spatial displacement along the symmetry axis within the unit cell. This displacement robustness has been experimentally verified. By introducing an asymmetry parameter (*α*) to controllably break the mirror symmetry while simultaneously disrupting local inversion symmetry, a controlled transition from BIC to QBIC is achieved, with the quality factor scaling as Q ∝ *α* ^−1.95^. Momentum‐space analysis reveals that this process is accompanied by the splitting of a topological vector singularity (*q* = +1) into a pair of circular polarization singularities with opposite handedness (*C*+/*C*−). Near‐field and multipole analyses further clarify how symmetry breaking opens radiative channels and disrupts the balance of multipole interference. Finally, experimental measurements successfully observed the evolution from no resonance to a sharp Fano resonance, validating the effectiveness of the design principle. This study demonstrates the synergistic roles of local inversion symmetry and mirror symmetry in BIC formation and stability, offering a strategy and paradigm for developing terahertz photonic devices with high fabrication robustness.

## 2 Results and Discussion

The SP‐BIC metasurface with in‐plane mirror symmetry is depicted in Figure 1a. This periodic structure consists of two metallic elliptical cylinders, referred to as EP1 and EP2, arranged in a hexagonal lattice with mirror symmetry. Under ideal symmetric conditions (*r*_1_ = *r*_3_ = 60 μm, *r*_2_ = *r*_4_ = 20 μm), the structure supports a nonradiative BIC mode, with lattice constant *a* = 280 μm and inter‐cylinder instance *l* = 120 μm. By introducing a minor‐axis difference Δ*r* = *r*_4_ − *r*_2_, the in‐plane symmetry is disrupted, enabling the transition from BIC to QBIC, as shown in Figure 1b. To facilitate quantitative analysis across structures with different absolute ellipse dimensions, the normalized asymmetry parameter *α* = Δ*r*/(*r*_2_ + *r*_4_) is introduced as the normalized form of Δ*r* and used to quantify the degree of symmetry breaking. When *α =* 0, the structure maintains perfect mirror symmetry, and BIC mode cannot couple to free space, showing no significant resonant feature in the transmission spectrum. A sharp Fano resonance develops at 1.1 THz for *α* > 0, which broadens and redshifts with increasing *α*. The band structure of the metasurface, shown in Figure 1c (see more details in the Supporting Information S1: Note S1), confirms the existence of an SP‐BIC mode at 1.0985 THz. Further quantitative analysis in Figure 1d shows that the Q‐factor of the QBIC decays with increasing *α*, strictly following a power‐law scaling relation of Q_*rad*_ ∝ *α* ^−1.95^ (see more details in the Supporting Information S1: Note S2). This precise scaling law confirms that the radiative loss of the mode can be quantitatively controlled via symmetry breaking. Therefore, by selectively breaking a specific mirror symmetry within the structure, the radiative channels of the QBIC can be directionally opened. This approach provides a solid physical foundation and a clear technical pathway for the inverse design of high‐performance, tunable metasurface devices.

**FIGURE 1:**
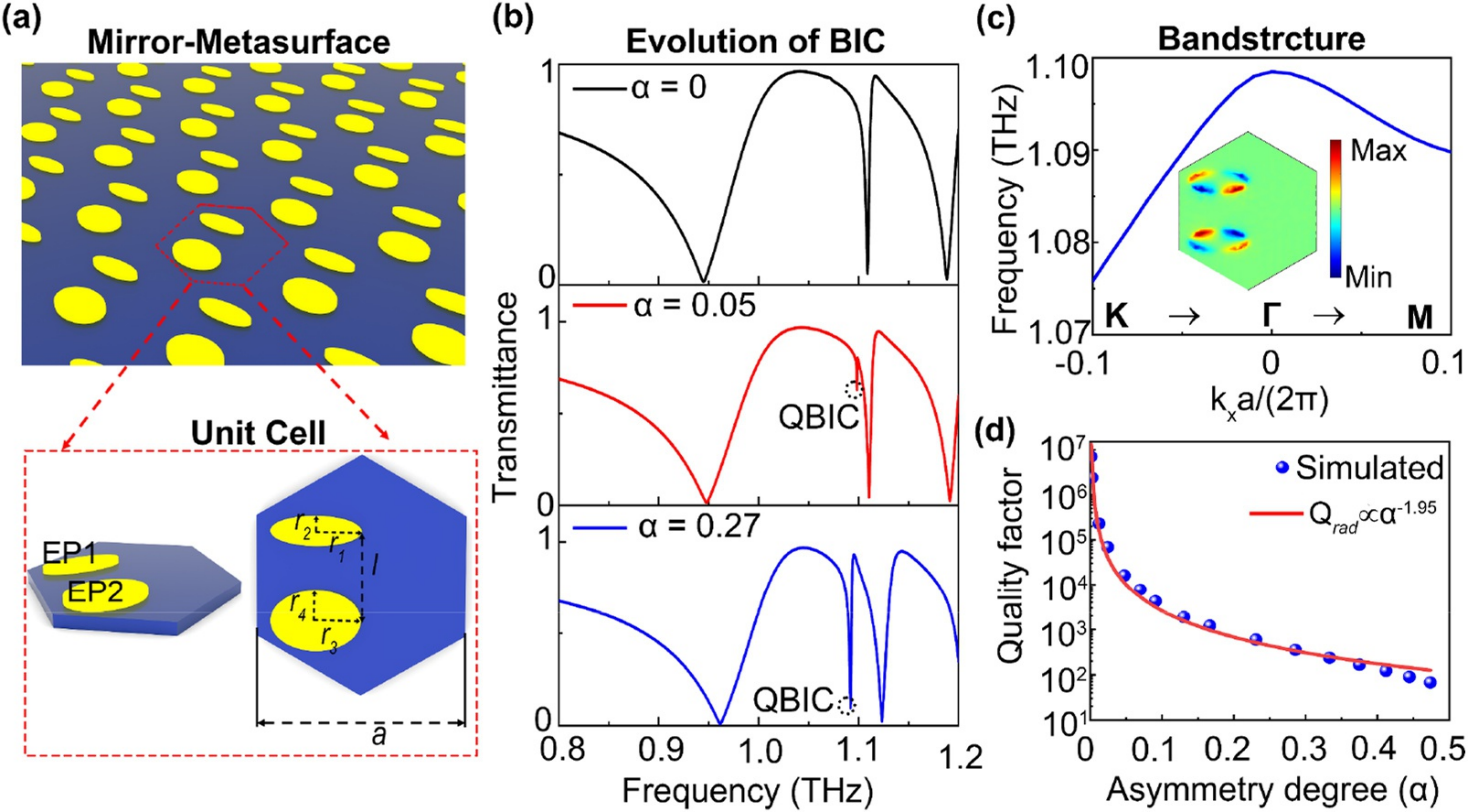
(a) Schematic of the metasurface, where EP1 and EP2 represent two metallic elliptical cylinders with semi‐major and semi‐minor axes defined as (*r*_1_, *r*_2_) and (*r*_3_, *r*_4_), respectively. The structural asymmetry is introduced by the short‐axis difference Δ*r* = *r*_4_ − *r*_2_, and the normalized asymmetry parameter is defined as *α* = Δ*r*/(*r*_2_ + *r*_4_). (b) Simulated transmittance spectra for three representative asymmetry parameters *α* = 0, 0.05, and 0.27. At *α* = 0, the structure possesses perfect mirror symmetry and the resonant mode is completely decoupled from free‐space radiation, resulting in a featureless transmission background characteristic of an ideal BIC with zero radiative leakage. As *α* increases, the mirror symmetry is broken and the BIC evolves into a radiatively accessible QBIC, manifesting as a sharp Fano resonance in the transmission spectrum. (c) Photonic band structure of the metasurface along the high‐symmetry path *K→Γ→M* in the first Brillouin zone at *α* = 0. The inset shows the *E*_z_ electric field distribution of the BIC mode at the *Γ*‐point. (d) Simulated radiative quality factor (Q_*rad*_) as a function of asymmetry degree *α*. Bilue dots represent simulation results extracted from complex eigenfrequencies via Q_*rad*_ = *Ω*/(2*γ*) (where *Ω* is the real part of the eigenfrequencies, and *γ* denotes the radiation loss). The red curve indicates the power‐law fitting Q_*rad*_ ∝ *α* ^−1.95^.

After confirming the regulatory capacity of mirror symmetry over radiative losses, we further explored the symmetry protection mechanism of this SP‐BIC. Figure 2a illustrates the transition from a structure with global inversion symmetry to one with local inversion symmetry. When the geometric center of the dimer (*O*_1_) coincides with the center of the hexagonal lattice (*O*) (the spatial offset Δ*d* = 0), the entire structure possesses global inversion symmetry, satisfying the coordinate transformation (*x*, *y*) = (−*x*, −*y*). At this stage, a polarization vortex center *V*_1_ appears in the structure at the corresponding frequency, with its topological charge calculated as $q=\frac{1}{2\pi }\oint_{C}dk\cdot \nabla_{k}\phi \left(k\right),q\in Ζ$. Here, *C* denotes an arbitrary closed‐up path encircling the BIC once counterclockwise in momentum space, and ϕ(k) represents the angle between the polarization vector and the *x*‐axis. The calculated topological charge is *q*_1_ = +1. When *O*_1_ and *O* do not coincide (Δ*d* ≠ 0), the global inversion symmetry of the structure is broken. Nevertheless, the overall structure retains in‐plane mirror symmetry about the *x*‐axis (satisfying (*x*, *y*) = (*x*, −*y*)), and the metallic elliptical pillar dimer itself preserves its local inversion symmetry. As Δ*d* increases from 0 to 60 μm, the distribution of far‐field polarization states and the topological properties of the mode remain essentially unchanged, as shown in Figure 2c–e. This finding demonstrates that the BIC mode fundamentally originates from the local inversion symmetry of the dimer, whereas the overall mirror symmetry of the structure provides robust protection, ensuring that even when global inversion symmetry is lost due to spatial offset, the bound state remains completely decoupled from the radiative continuum. This behavior stands in direct contrast to conventional global‐inversion‐symmetric designs, where an equivalent dimer displacement would immediately break the inversion symmetry of the entire unit cell, open a radiative channel, and degrade the BIC into a leaky resonance.

**FIGURE 2:**
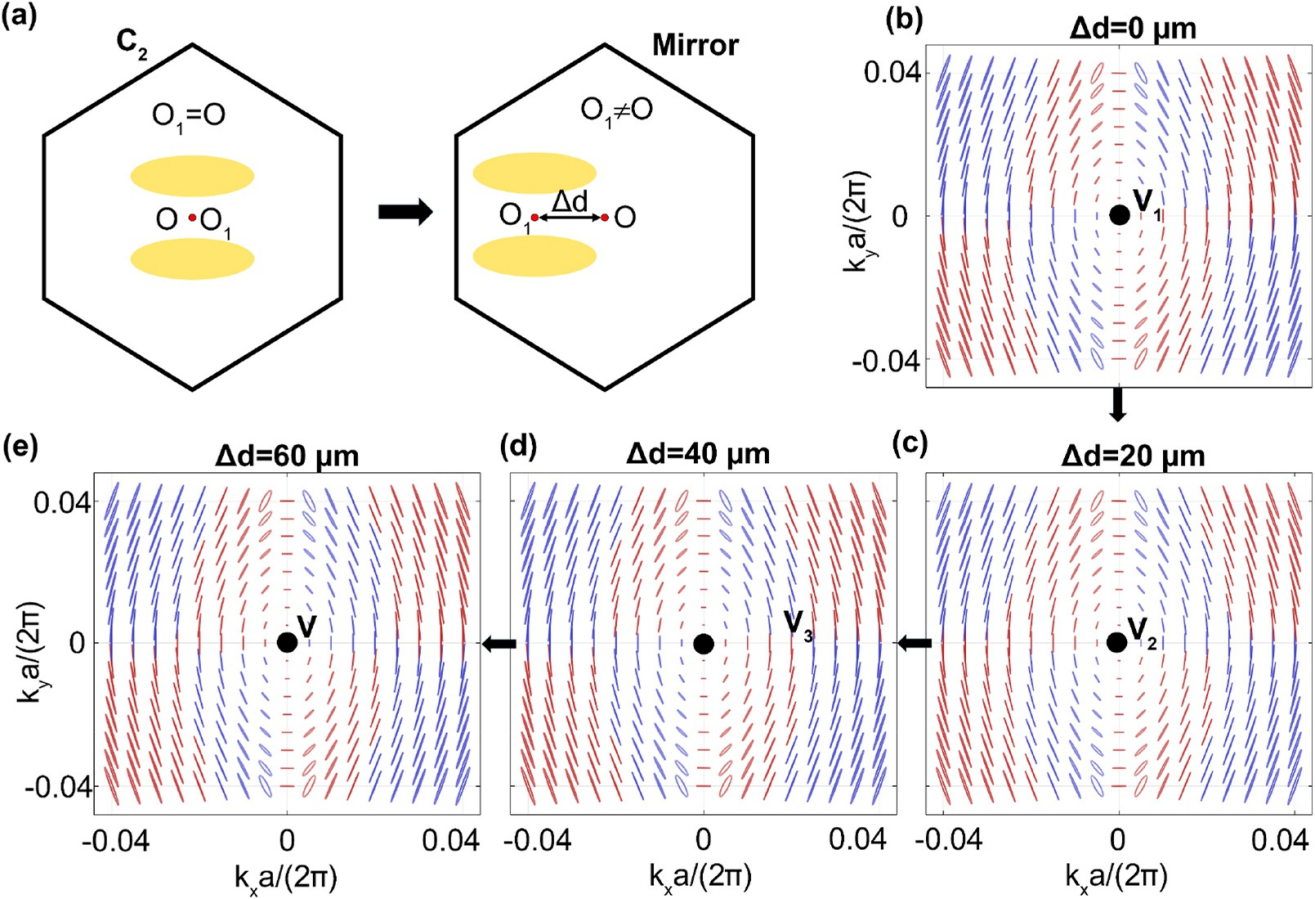
(a) Schematic of the unit cell transitioning from global inversion symmetry (Δ*d* = 0) to a configuration with only mirror symmetry (Δ*d* ≠ 0). *O* and *O*_1_ denote the centers of the hexagonal lattice and the dimer, respectively, with Δ*d* representing the offset distance. (b)–(e) Far‐field polarization distributions for different offsets: (b) Δ*d* = 0 μm, (c) Δ*d* = 20 μm, (d) Δ*d* = 40 μm, and (e) Δ*d* = 60 μm. The polarization singularity at the *Γ*‐point (*V*‐point, topological charge *q* = +1) remains invariant despite the breaking of global *C*_2_ symmetry. This demonstrates that the BIC mode, while fundamentally rooted in local symmetry, exhibits exceptional robustness against spatial displacements under the protection of mirror symmetry.

The topological origin and momentum‐space evolution of the BIC are further investigated using the aforementioned platform. As shown in Figure 3c, a polarization vortex center *V* emerges at the *Γ*‐point in momentum space under perfect symmetry (*α* = 0, Δ*r* = 0). Correspondingly, no resonant feature is observed in the transmittance spectrum (Figure 3a), signifying the complete closure of radiative channels and manifesting as an ideal BIC. When an asymmetry parameter is introduced (*α* = 0.11, Δ*r* = 5 μm), the structural symmetry is broken, as shown in Figure 3d. This vector singularity (*V*) splits into a pair of circular polarization singularities with opposite topological charges: a left‐handed (*C*+) and a right‐handed (*C*−) circular polarization singularity (see more details in Supporting Information S1: Note S3). This pair of circular polarization singularities becomes separated in momentum space. Concurrently, in the transmittance spectrum (Figure 3b), a sharp, high‐Q Fano resonance appears where none existed before, marking the transition from a BIC to a QBIC. This result clearly demonstrates that the splitting of topological singularities in momentum space and the radiative leakage in real space (i.e., the emergence of the QBIC) are manifestations of the same physical process across different dimensions.

**FIGURE 3:**
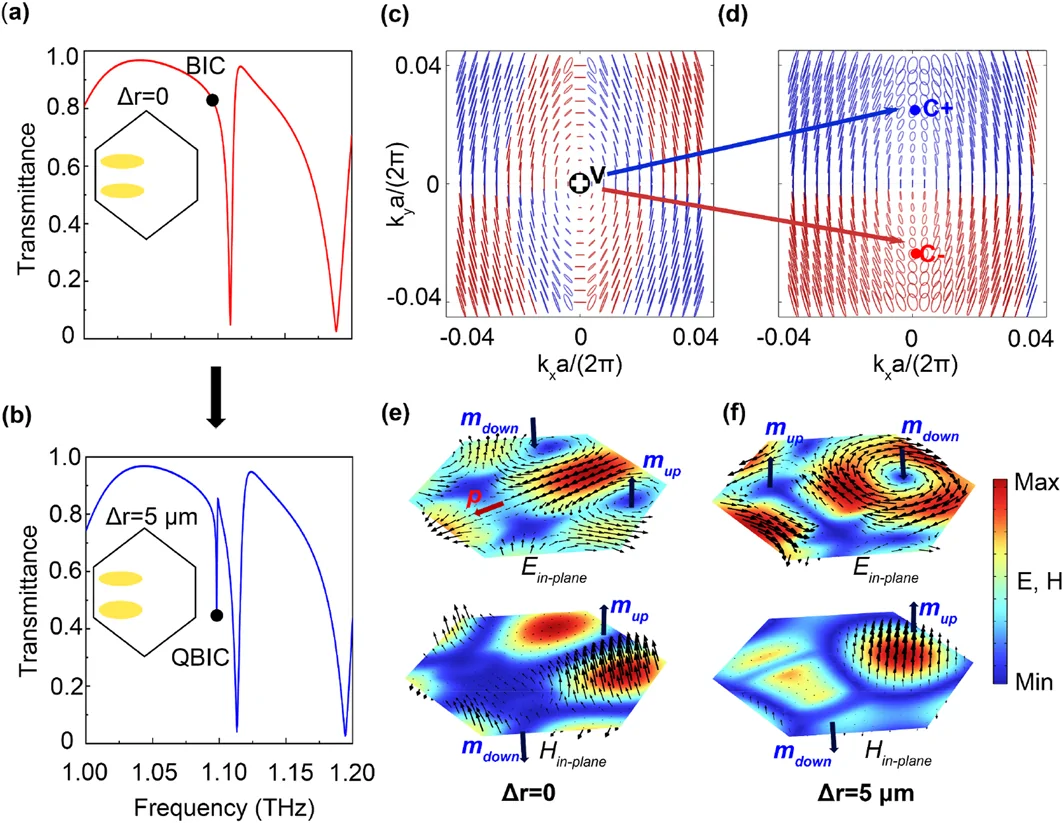
(a), (b) The transmittance spectra under normal incidence at Δ*r* = 0 and Δ*r =* 5 μm. The insets show the schematic of the structure for the respective parameters. (c), (d) Evolution of far‐field polarization singularities in momentum space. (c) Under perfect symmetry (Δ*r* = 0 μm, *α* = 0), a vector singularity with a topological charge of *V* = +1 appears in the far‐field polarization distribution. (d) When structural asymmetry is introduced (Δ*r* = 5 μm, *α* = 0.11), this vector singularity splits into a pair of circular polarization singularities: left‐handed (*C*+) and right‐handed (*C*−). (e), (f) In‐plane electric field *E*_in‐plane_ and magnetic field *H*_*in‐plane*_ distributions, along with the corresponding electric (***p***) and magnetic (***m***) dipole moment distributions, for the cases of Δ*r* = 0 and Δ*r* = 5 μm, respectively.

The near‐field physical mechanisms underlying the far‐field responses are elucidated through an in‐depth analysis of the in‐plane electromagnetic field distribution. As illustrated in Figure 3e, in the perfectly symmetric BIC state (*α* = 0), the near‐field is dominated by the in‐plane electric dipole moment (***p***) and the electric quadrupole moment, with ***p*** aligned along the *x*‐direction. The strict mirror symmetry of the structure causes complete destructive interference between their far‐field radiations, whereas the magnetic dipole (MD) moment cancels to zero as the upper and lower components oppose one another (***m***_*up*_ = −***m***_*down*_). This collective suppression of multipolar radiation perfectly confines the mode within the structure. However, when *α* > 0 (Figure 3f), the structural distortion disrupts this precise multipolar balance: the emergence of a net magnetic dipole opens an initial radiation channel. More critically, symmetry breaking excites a strong toroidal dipole (TD) response, which becomes the dominant radiation source and transforms the BIC into an observable high‐Q QBIC.

The subsequent multipole scattering analysis (Figure 4a–c) provides quantitative evidence for the physical concepts [31, 32, 33, 34, 35, 36] (see more details in Supporting Information S1: Note S4). The scattering power spectra confirm that at *α* = 0, the contributions from the magnetic dipole, magnetic quadrupole, and toroidal dipole are suppressed to near zero. The remaining electric dipole and electric quadrupole scatterings exhibit a phase difference of 3.14 rad (approximately π) at 1.0985 THz (Figure 4d). This anti‐phase characteristic, close to 180°, quantitatively confirms the mechanism of complete destructive interference in the far‐field radiation (Figure 4f). When asymmetry is introduced (*α* = 0.11), the scattering response undergoes a fundamental transformation, as shown in Figure 4b. In this state, the phase balance is significantly disrupted, and the relative phase between the ED and EQ deviates from π (Figure 4e,f). At the same time, the scattering power of the previously confined TD grows explosively, becoming the dominant radiation component. The scattering power of other multipoles, such as the MD and EQ, also increases markedly. This systematic enhancement of various multipolar orders, revealed from the perspective of energy scattering, aligns closely with the conclusions of the near‐field analysis, conclusively revealing the physical essence of how symmetry governs the radiative loss of the BIC.

**FIGURE 4:**
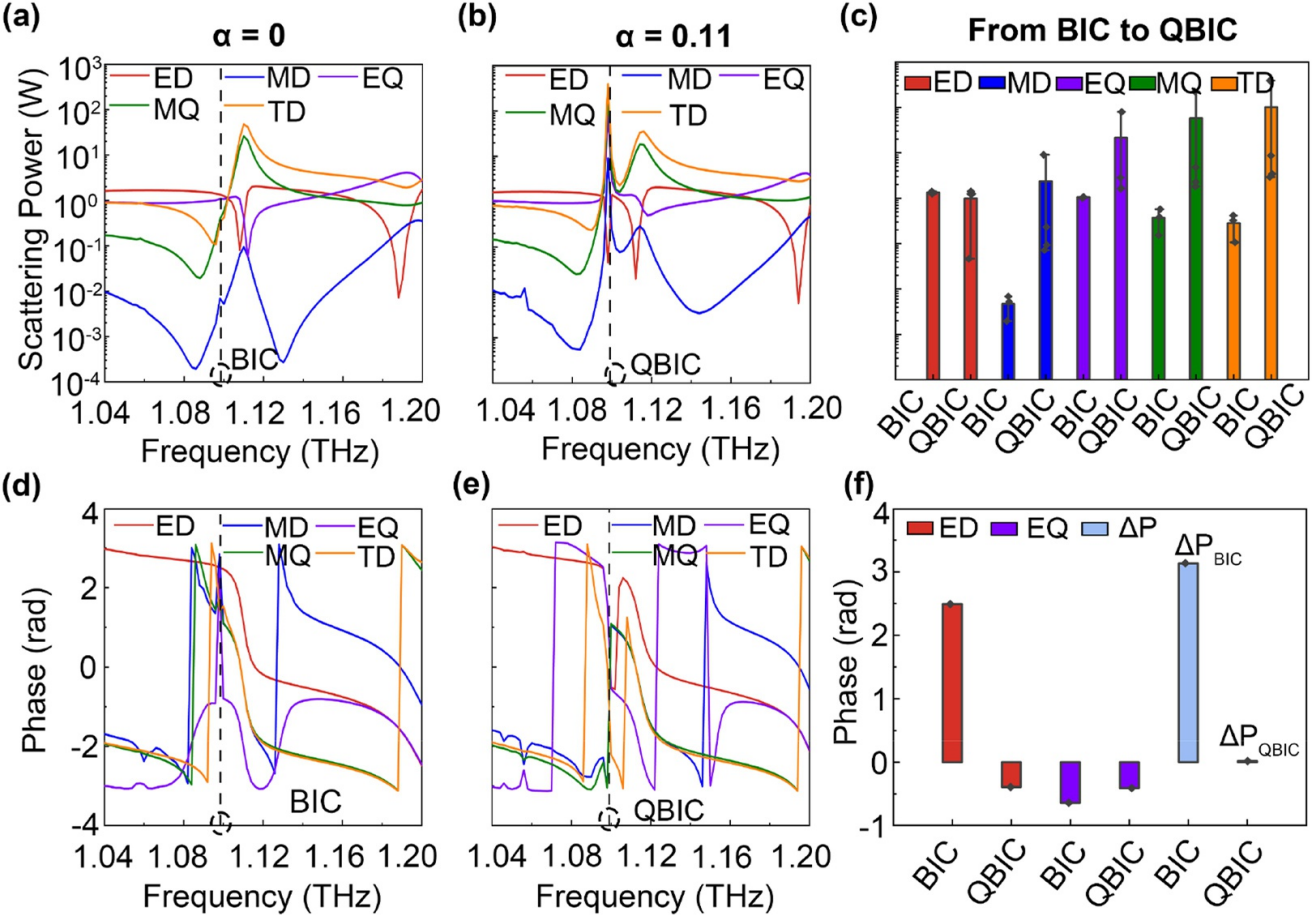
(a) The multipole scattering spectra under the BIC mode. (b) The multipole scattering spectra under the QBIC mode (*α* = 0.11). Here, ED is the electric dipole, MD is the magnetic dipole, EQ is the electric quadrupole, MQ is the magnetic quadrupole, and TD is the toroidal dipole. (c) Quantitative comparison of the multipole scattering power. The bar chart compares the distribution of scattering power for each multipole at their respective characteristic frequencies in the BIC and QBIC states. (d) Phase spectra of multipolar coefficients for the perfectly symmetric metasurface (*α* = 0). The vertical dashed line indicates the ideal BIC frequency at 1.0985 THz. (e) Phase spectra of multipolar coefficients for the asymmetric metasurface with *α* = 0.11. The vertical dashed line marks the resonance frequency of the QBIC mode at 1.0990 THz. (f) Quantitative comparison of the phases and relative phase differences between the ED and EQ at the BIC and QBIC frequencies. At the BIC frequency, the phase difference (Δ*P*_BIC_) is approximately 3.14 rad (≈π). In the QBIC state, this phase balance is disrupted (Δ*P*_QBIC_), leading to radiative leakage.

Theoretical and simulation studies have shown that the BIC observed in this structure arise from local inversion symmetry, whereas the overall mirror symmetry of the structure ensures robustness. To validate the feasibility of this mechanism in practical devices and to quantify its performance, we designed and fabricated a series of flexible metasurface samples. As shown in Figure 5a, the sample structures are classified into three typical configurations based on the asymmetry parameter *α* (corresponding to *α* = 0, 0.27, 0.33), with detailed geometric dimensions listed in Table 1. The transmission spectra measured using a terahertz time‐domain spectroscopy (THz‐TDS) system [37] clearly reveal the evolution from a nonradiative SP‐BIC to a radiative QBIC (see Supporting Information S1: Note S5 for details). As illustrated in Figure 5b,c, under the perfectly symmetric conditions (*α* = 0), no resonance peak appears near 1.1 THz in the experimental and simulated spectra, directly confirming the nonradiative nature of the ideal BIC. With increasing asymmetry (*α* = 0.27, 0.33), sharp transmission dips gradually emerge and deepen in the spectra, marking the controlled leakage of bound‐state energy into free space. The deepening of these transmission dips intuitively reflects the increasing radiative loss as *α* increases, which is highly consistent with simulation predictions. Quantitative analysis was performed by fitting the measured and simulated transmission spectra with the Fano formula (see Supporting Information S1: Note S6 for details), from which the total Q‐factors (Q_total_) are extracted. To enable a meaningful comparison with the experiment, the simulations in Figure 5c incorporate the experimentally reported complex permittivity of the polyimide substrate (*ε* = 3.5, tan*δ* = 0.0027) [38], so that the simulated Q_total_ values account for both radiative and material dissipation losses. The simulated Q_total_ values at *α* = 0.27 and 0.33 are 230.17 and 159.56, respectively, whereas the experimentally extracted values are 87.82 and 80.24, respectively. Although the experimental values are substantially lower than the simulated ones, their evolution trend—decreasing as the asymmetry parameter *α* increases—remains highly consistent, qualitatively verifying the physical law that the radiative loss of the QBIC is regulated by symmetry breaking. The discrepancy in magnitude primarily stems from nonideal factors in the experimental environment [39, 40, 41]. First, dimensional deviations and edge roughness inherent to the photolithography and wet‐etching process introduce additional scattering losses. Second, batch‐to‐batch variations in the copper film quality and polyimide substrate uniformity—arising from outsourced fabrication—contribute to sample‐to‐sample differences in ohmic and dielectric losses. Third, supplementary simulations under oblique‐incidence excitation (cone half‐angle up to 5°) reveal a pronounced sensitivity of the QBIC resonance to the incident angle that goes well beyond a minor perturbation: as the angle *θ* increases from 0° to 5°, the extracted Q‐factor decreases from 230.17 to 55.34 for *α* = 0.27 and from 159.56 to 83.03 for *α* = 0.30, accompanied by a progressive blueshift of the resonance frequency (see Supporting Information S1: Note S7 for details). Because the focused THz beam in the experiment inevitably contains a finite cone of propagation directions, the measured spectrum integrates contributions from this range of incident angles, resulting in substantial inhomogeneous broadening of the resonance linewidth and a corresponding large reduction in the experimentally extracted Q‐factor. Moreover, the limited spectral resolution of the THz‐TDS system hinders the detection of subtle center‐frequency shifts, especially given the broad spectral envelope of low‐Q resonances.

**FIGURE 5:**
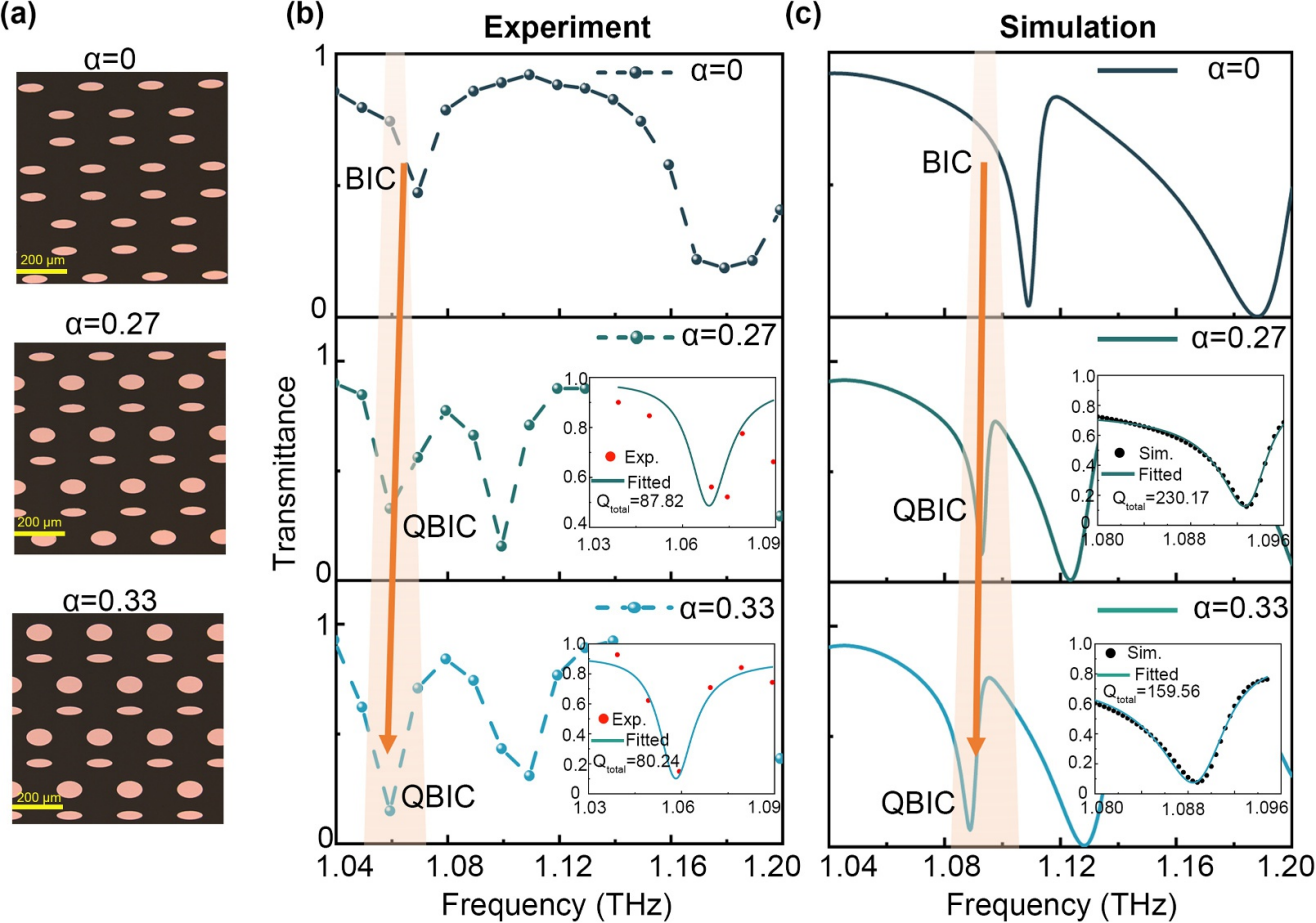
(a) SEM images of the fabricated metasurface samples for *α* = 0, 0.27, and 0.33, respectively. (b) Experimental transmission spectra and (c) simulated transmission spectra of the metasurface for *α* = 0, 0.27, and 0.33, respectively. The simulations in (c) incorporate the measured complex permittivity of the polyimide substrate (*ε* = 3.5, tan*δ* = 0.0027), yielding total Q‐factors (Q_total_) that account for both radiative and material dissipation losses. The insets in (b) and (c) show enlarged views of the Fano resonance profiles near the QBIC frequency, with the solid curves representing fits to the Fano formula, from which the total quality factor (Q_total_) is extracted.

**TABLE 1:** Comparison of the structural parameters of fabricated samples and simulated samples.

Samples		*a* [um]	*r*_1_ [um]	*r*_2_ [um]	*r*_3_ [um]	*r*_4_ [um]	*l* [um]
1	Sim.	280	60	20	60	20	120
	Exp.	281.51	60.68	20.65	61.06	20.21	120.65
2	Sim.	280	60	20	60	35	120
	Exp.	281.50	61.10	21.05	60.63	36.63	121.25
3	Sim.	280	60	20	60	40	120
	Exp.	282.36	61.11	21.05	61.13	40.85	122.101

Overall, the high consistency between simulation and experiment not only confirms the accuracy of our method for constructing two‐dimensional SP‐BIC metasurfaces and the validity of the theoretical framework but also underscores the significant role of the asymmetry parameter *α* in modulating optical properties, including the Q‐factor, resonance frequency, and radiation efficiency. This mirror‐symmetry‐based strategy for realizing highly robust BIC devices avoids the strict constraints imposed by complex global symmetries, offering a technological avenue for high‐performance terahertz sensors and integrated photonic devices with high fabrication tolerance.

## 3 Conclusion

In summary, this study successfully demonstrates that a dimer structure with local inversion symmetry can support an offset‐robust SP‐BIC in a hexagonal lattice metasurface, provided in‐plane mirror symmetry is preserved. This mode remains robust at the *Γ*‐point even when global periodic inversion symmetry is broken by dimer displacement, substantially relaxing fabrication precision requirements. By introducing a single geometric asymmetry parameter *α*, defined as the half‐minor axis difference Δ*r/|r*_2_ + *r*_4_ *|*, the work achieves controlled evolution from an ideal BIC to a QBIC. Its Q‐factor follows a power‐law scaling relationship of Q ∝ *α* ^−1.95^, quantitatively revealing the tuning mechanism of radiative loss. Momentum‐space analysis and multipole decomposition further indicate that this process is accompanied by the splitting of the topological charge singularity with *q* = +1, along with disruption of destructive interference among the electric dipole, electric quadrupole, and toroidal dipole due to symmetry breaking. The strong agreement between experimental and simulation results confirms that local inversion symmetry is the physical origin of BIC formation, while also underscoring the critical role of mirror symmetry in enabling fabrication tolerance. This work provides a practical theoretical framework and design guidelines for developing high‐performance, highly reliable terahertz integrated photonic devices.

## 4 Experimental Section

The experiments were conducted using a THz‐TDS system. The system primarily comprises a femtosecond laser, terahertz emission and detection units, an optical delay line, and associated mirrors and detectors. THz‐TDS is a coherent detection technique that simultaneously acquires both amplitude and phase information from the transmitted signal. Its core operating principle is as follows: a femtosecond laser pulse excites a photoconductive antenna, generating a broadband terahertz pulse, which is then split by a beam splitter into two paths. One serves as the probe terahertz pulse that transmits through the sample under test perpendicularly, carrying spectral response information before being received by the terahertz detector. The other acts as the reference laser pulse, which passes through an optical delay line precisely controlled by a stepper motor and enables gated sampling of the terahertz pulse at the detector. By varying the delay time, the complete time‐domain waveform of the terahertz pulse can be obtained.

## 5 Sample Fabrication

The metallic structures are copper (Cu) thin films with a nominal thickness of 200 nm (tolerance ± 10 nm), deposited by magnetron sputtering at a base vacuum pressure of ∼10^−4^ Pa. A ∼10 nm titanium (Ti) adhesion layer was inserted between the Cu film and the polyimide (PI) substrate to enhance interfacial adhesion. The complete fabrication procedure is as follows. All device units were processed on a 500 μm‐thick SiO_2_ carrier wafer. After ultrasonic cleaning, a 20 μm‐thick PI precursor film was spin‐coated onto the wafer and thermally cured at 300°C to form the PI substrate. A photoresist layer was then spin‐coated and soft‐baked to remove residual solvent. The resist was subsequently patterned by UV exposure through a chrome photomask and developed to remove the unexposed regions. A Ti (10 nm)/Cu (200 nm) bilayer was then deposited by magnetron sputtering. Finally, the metal pattern was defined by a lift‐off process in which the photoresist and the overlying metal film were dissolved in chemical solvent, leaving only the desired metallic structures on the PI film. The fabricated samples were then peeled from the carrier wafer to yield free‐standing flexible metasurface devices.

## Author Contributions

**Die Zou:** data curation, investigation, methodology, writing – original draft, formal analysis. **Zhen Zhou:** conceptualization, investigation, formal analysis, methodology. **Xia Cao:** formal analysis, methodology. **Rui Zhu:** formal analysis, software. **Keyu Tan:** investigation, methodology. **Zhiyong Wang:** project administration, supervision. **Xiaohua Xing:** conceptualization, supervision, validation. **Jianquan Yao:** supervision, validation, writing – review and editing. **Jianing Chen:** conceptualization, visualization, supervision. **Liang Wu:** conceptualization, funding acquisition, project administration, supervision, writing – review and editing.

## Conflicts of Interest

The authors declare no conflicts of interest.

## Supporting information


Supporting Information S1

## Data Availability

The data that support the findings of this study are available from the corresponding author upon reasonable request.
