# Supplementary material for: Robust Bound States in the Continuum in Mirror‐Symmetric Terahertz Metasurfaces
Source: Nanophotonics. 2026 Jul 17;15(14):e70221. doi: 10.1002/nap2.70221 (PMC13642053; doi:10.1002/nap2.70221)
Supplement: Supplementary file 1 [file NAP2-15-e70221-s001.pdf]

Supporting Information

Robust Bound States in the Continuum in Mirror-Symmetric Terahertz Metasurfaces

Die Zou[1], *Zhen Zhou*[1], *Xia Cao*[1], *Rui Zhu*[1], *Keyu Tan*[2], *Zhiyong Wang*[2], *Xiaohua Xing*[1], *Jianquan Yao*[1], *Jianing Chen*[3], and *Liang Wu*[1,*]

[1] College of Precision Instrument and Optoelectronics Engineering, Tianjin University, Key Laboratory of Optoelectronics Information and Technology (Ministry of Education), Tianjin, 300072, China | [2] College of Mechanical Engineering, Tianjin University, Key Laboratory of Modern Engineering Mechanics (Ministry of Education), Tianjin, 300072, China | [3] The Institute of Physics, Chinese Academy of Sciences & School of Physical Sciences, University of Chinese Academy of Sciences, Beijing 100190, China

Correspondence: Liang Wu (wuliang@tju.edu.cn)

Content

Supporting Note S1. Band structure calculation of a hexagonal lattice 2
Supporting Note S2. The solution of the scaling law 3
Supporting Note S3. Symmetry-breaking-induced splitting of topological singularities and *C*-point evolution 4
Supporting Note S4. Multipole decomposition 5
Supporting Note S5. Experimental procedure 6
Supporting Note S6. Quantitative fitting and parameter characterization of QBIC modes 7
Supporting Note S7. Numerical evaluation of experimental non-idealities on QBIC Q-factor degradation 8

Supporting Note S1. Band structure calculation of a hexagonal lattice

Figure S1 illustrates the real-space geometry and corresponding reciprocal space of the two-dimensional hexagonal lattice metasurface used in this study. The first Brillouin zone (FBZ) of this periodic system is a regular hexagon. To fully characterize the dispersion evolution of the SP-BIC and its QBIC mode, we computed the band structure along the high-symmetry path *K* to *Γ* to *M* in reciprocal space. Since the BIC mode resides at the BZ center, this path clearly reveals the bandgap and polarization topological evolution near the *Γ*-point.

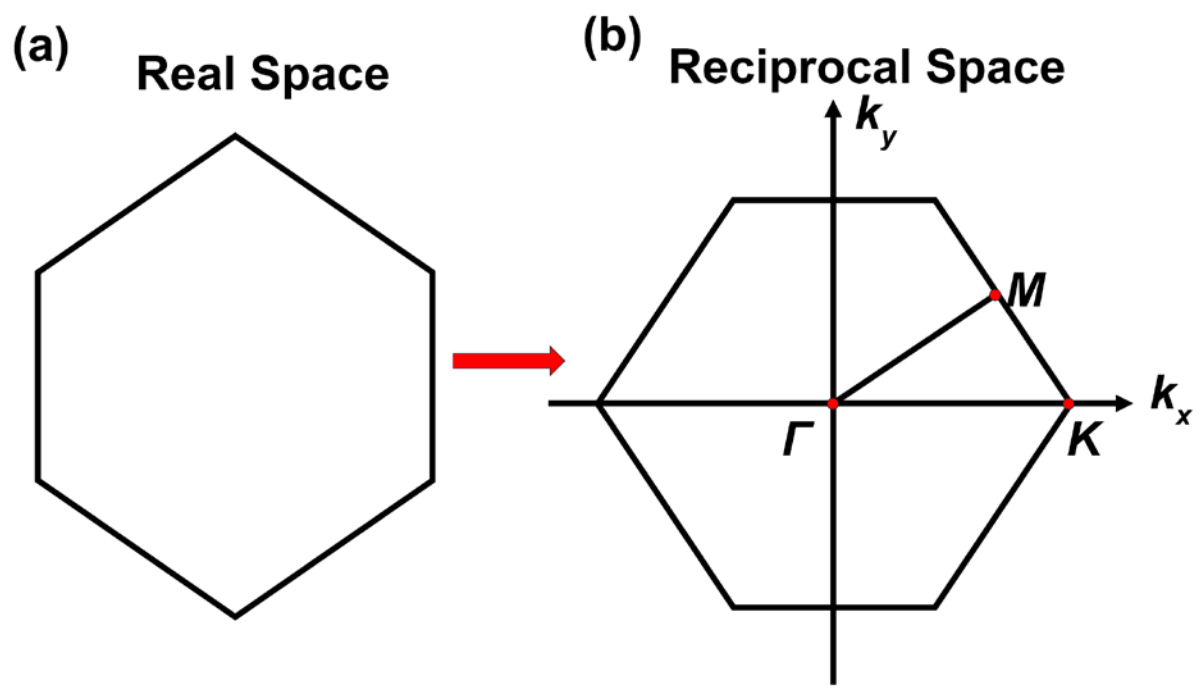

Figure S1. Schematic of a two-dimensional hexagonal lattice and its corresponding reciprocal space. (a) Real-space representation. (b) Reciprocal-space representation, with the first Brillouin zone of the hexagonal lattice and the high-symmetry points *Γ*, *K*, and *M* indicated.

To enable a continuous sweep along the folded *K* to *Γ* and *Γ* to *M* paths in the COMSOL Multiphysics eigenfrequency solver, we adopted a one-dimensional parameterized mapping strategy. A normalized sweep parameter k was defined over the range [-0.1, 0.1], with k=0 fixed at the *Γ*-point at the center of reciprocal space. The intervals k∈[-0.1, 0) and k∈(0, 0.1] correspond to local momentum-space trajectories along the *K* to *Γ* and *Γ* to *M* directions, respectively. Through piecewise functions and coordinate transformations tied to the lattice constant, the system converts the one-dimensional sweep parameter k into two-dimensional wavevector coordinates (k_x, k_y). This dimension-reduced sweeping method avoids the computational cost of multi-dimensional parameter sweeps and ensures that eigenvalue calculations remain strictly on the intended high-symmetry path in momentum space. Based on the mapping logic described above, the wavevector components k_x and k_y were configured in the COMSOL Global Definitions (Parameters and Variables) as follows:

k0	4*pi/(a*sqrt(3))
alpha	if(k<=0, -2*k/3, 0.5*k)
beta	if(k<=0, -k/3, 0.5*k)
k_x	alpha*k0*sqrt(3)/2
k_y	k0*(beta-0.5*alpha)

Supporting Note S2. The solution of the scaling law

Through the eigenfrequency solver of COMSOL Multiphysics, we extracted the complex eigenfrequencies $\omega=\Omega-i\gamma$ for QBIC modes under varying asymmetry parameters α. The real part Ω denotes the resonant angular frequency, and the imaginary part γ quantifies the total radiative decay rate of the system. It should be noted that the substrate material was set as lossless in the simulations. Therefore, γ reflects only the radiative decay of the structure and does not include material absorption losses. The Q-factor of the corresponding structures was then calculated using $Q=\Omega/(2\gamma)$.

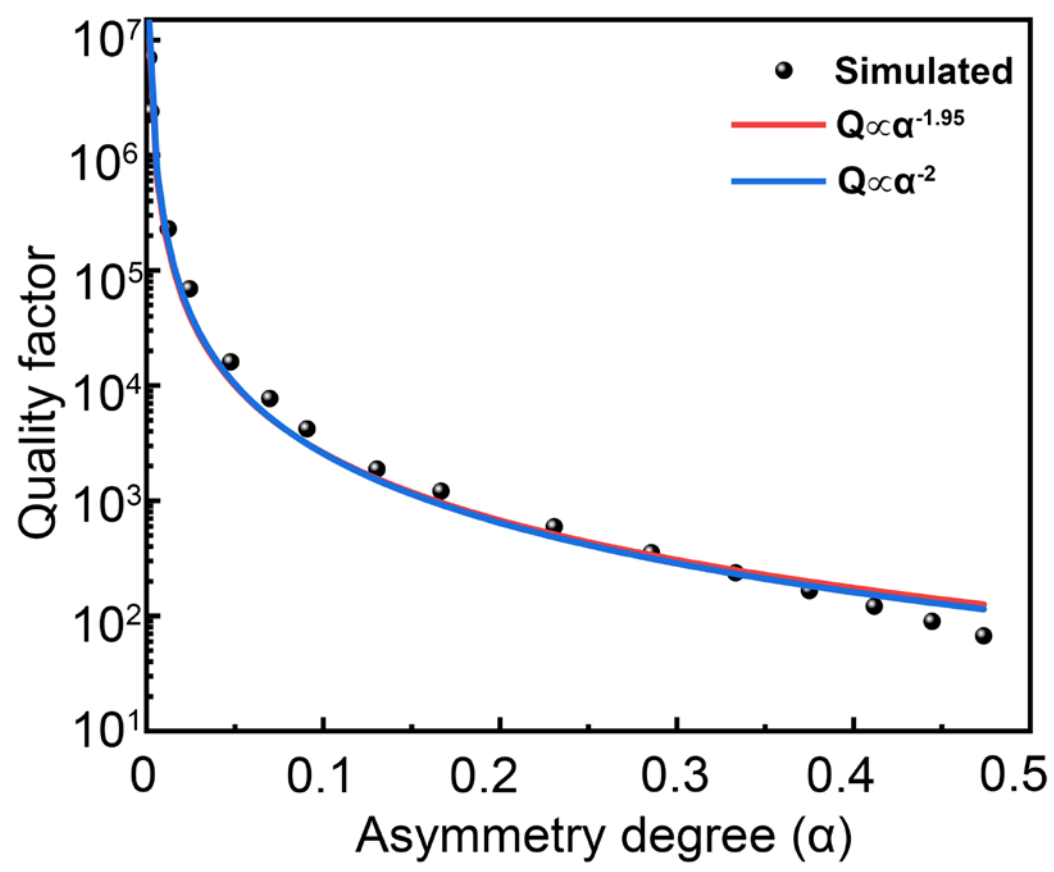

Figure S2. Scaling of the radiative quality factor (Q_{rad}) with the asymmetry parameter α. Blue dots represent the simulated Q_{rad}, extracted from the complex eigenfrequencies of the COMSOL eigenfrequency solver via $Q_{rad}=\Omega/(2\gamma)$, where Ω is the real part of the eigenfrequency and γ is the radiative decay rate. The red solid curve represents the best power-law fit, yielding an exponent of $n\approx1.95$. The black dashed line indicates the theoretical inverse-square scaling with $n=2$, expected for an ideal SP-BIC. The slight deviation from $n=2$ is attributed to higher-order multipole contributions and the finite size of the simulation domain.

To examine the quantitative scaling of radiative leakage in this mode, we fitted a power-law function ($Q=A\alpha^{-n}$) to the (α, Q) dataset in MATLAB. The optimal fitting exponent was found to be $n\approx1.95$, closely matching the classic inverse-square scaling law ($Q\propto\alpha^{-2}$) derived from perturbation theory for SP-BICs. To further assess the applicability of the theoretical model, we compared two fitting strategies (shown in Figure S2): Model A (free fitting, with n treated as a free parameter) and Model B (theoretically constrained fitting, with n fixed at 2). The residual sum of squares (RSS) for Model A (0.417) was slightly lower than that for Model B (0.444), indicating a marginally better fit for the simulated data under the free-fitting approach.

We attribute this minor and reasonable deviation (Δn=0.05) to two key factors. First, the quadratic scaling law is derived under the strict perturbation limit of infinitesimal asymmetry (α to 0); within the finite α range investigated here, nonlinear radiation coupling effects from higher-order perturbation terms emerge, causing a slight departure from the ideal inverse-square law. Second, discretization of the simulation mesh and inherent uncertainties in fitting high-Q values introduce minor numerical variations. To quantify this difference on a statistical level, we calculated the standard error of the exponent n from the free-fitting model, with a confidence interval of approximately ±0.06. This implies that the theoretically predicted value (n=2) falls entirely within the statistical uncertainty window of our extracted value (n=1.95±0.06). This rigorous statistical evidence conclusively confirms the high physical consistency between our results and the classic scaling law, thereby providing strong support for the core physical picture: the opening of radiation channels dominated by local symmetry breaking follows an inverse-square relationship.

Supporting Note S3. Symmetry-breaking-induced splitting of topological singularities and *C*-point evolution

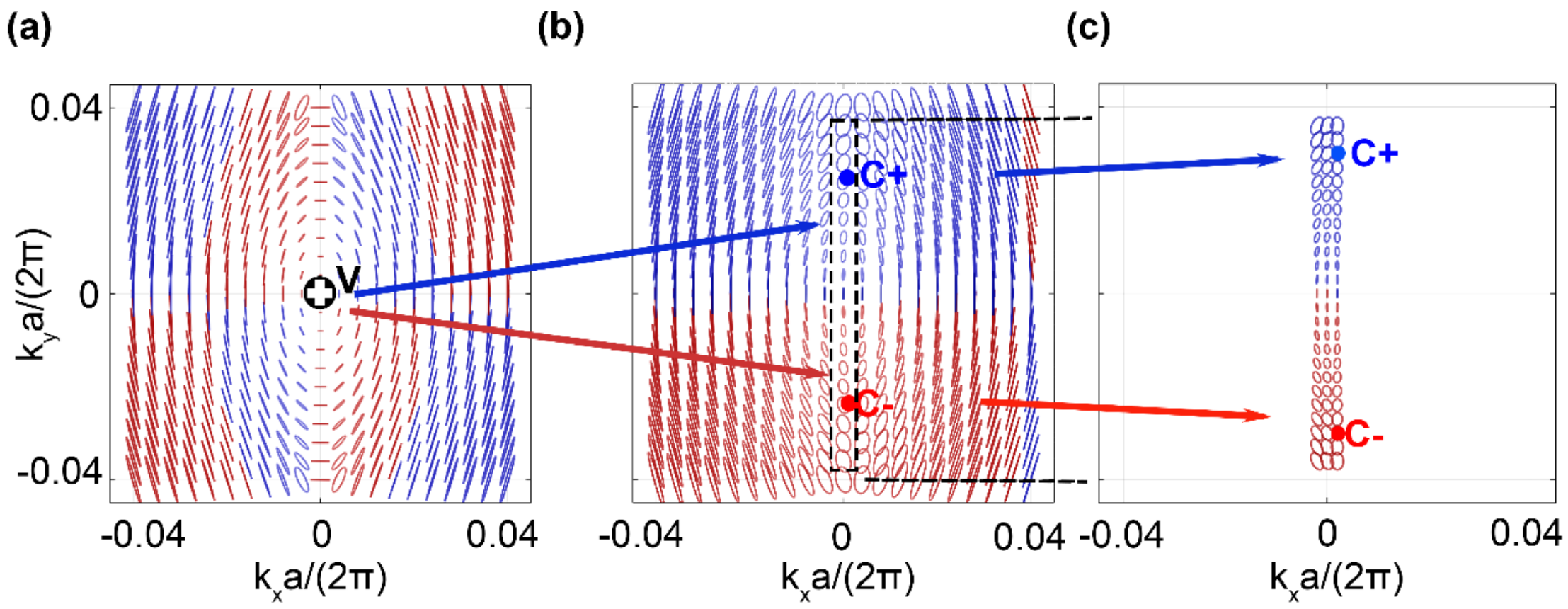

Figure S3. Topological evolution of polarization singularities in momentum space. Panels (a)-(c) illustrate the topological transition in the far-field polarization distribution of symmetry-protected bound states in the continuum as symmetry is broken. Figure (a) shows a vector singularity in a perfectly symmetric structure. In (b), symmetry breaking causes the singularity to split. Circular polarization singularities (*C*-points) appear in (c), obtained by extracting a subsection of the region shown in (b) and performing a refined simulation.

As shown in Figure S3, the color mapping represents the ellipticity of the far-field polarization states, which is a key metric for identifying circular polarization singularities (*C*-points), where

the ellipticity reaches ±1. To precisely locate the *C*-points, we calculated the ellipticity distribution across momentum space by extracting the Stokes parameters. Initial calculations indicated that following symmetry breaking (α>0), the extreme values of ellipticity are concentrated within a tiny region around $k_y a/(2\pi) \approx \pm 0.028$ (as shown in Figure S3b). However, due to the insufficient sampling density of the original grid, these localized features were spatially aliased, rendering the singular structures visually indistinguishable.

To resolve this, we implemented a high-resolution refinement strategy specifically targeting the suspected singular regions. By performing a fine-grained sweep (ranging from -0.036 to 0.036 for $k_y a/(2\pi)$ with a step of 0.003, and -0.002 to 0.002 for $k_x a/(2\pi)$ with a step of 0.002), the plotting resolution was significantly optimized. The refined Figure S3(c) clearly reveals a pair of circular polarization singularities with opposite helicities (*C*+ and *C*-). This observation provides a direct visual confirmation of the topological evolution during the QBIC excitation: the broken symmetry lifts the polarization degeneracy at the Γ-point, causing the original vector singularity (*V*-point) with a topological charge of q=+1 to split into a pair of *C*-points with opposite topological charges. This transition not only adheres to the principle of topological charge conservation but also offers robust topological evidence for the symmetry-governed radiative tuning mechanism proposed in this work.

Supporting Note S4. Multipole decomposition

To quantitatively analyze the significant roles of individual multipoles at the two BICs, the far-field scattering energies produced by the metasurface structure are calculated, compared, and evaluated. The multipole expansion in the Cartesian coordinate is used to explore the impact of the QBICs on the far-field. The multipole moments can be written as follows

Electric dipole (ED) moment:

$$\boldsymbol{P} = \frac{1}{i\omega}\int \boldsymbol{j}\, d^3\boldsymbol{r} \tag{S1}$$

Magnetic dipole (MD) moment:

$$\boldsymbol{M} = \frac{1}{2c}\int (\boldsymbol{r}\times\boldsymbol{j})\, d^3\boldsymbol{r} \tag{S2}$$

Electric quadrupole (EQ) moment:

$$\boldsymbol{Q}^{(e)}_{\alpha\beta} = \frac{1}{2i\omega}\int\left[r_\alpha j_\beta + r_\beta j_\alpha - \frac{2}{3}(\boldsymbol{r}\cdot\boldsymbol{j}) d^3\boldsymbol{r}\right] \tag{S3}$$

Magnetic quadrupole (MQ) components:

$$\boldsymbol{Q}^{(m)}_{\alpha\beta} = \frac{1}{3c}\int\left[(\boldsymbol{r}\times\boldsymbol{j})_\alpha r_\beta + (\boldsymbol{r}\times\boldsymbol{j})_\beta r_\alpha \right] d^3\boldsymbol{r} \tag{S4}$$

Toroidal dipole (TD) moment:

$$\boldsymbol{T} = \frac{1}{10c}\int\left[(\boldsymbol{r}\cdot\boldsymbol{j})\boldsymbol{r} - 2r^2\boldsymbol{j}\right] d^3\boldsymbol{r} \tag{S5}$$

where c is the speed of light, ω is the angular frequency, $\boldsymbol{j}$ is the current density displacement and $\boldsymbol{r}$ is the distance vector. The distribution of these moments is calculated via:

$$I_P = \frac{2\omega^4}{3c^3}\left|\boldsymbol{P}\right|^2 \tag{S6}$$

$$I_M = \frac{2\omega^4}{3c^3}\left|\boldsymbol{M}\right|^2 \tag{S7}$$

$$I_{Q^{(e)}} = \frac{\omega^6}{5c^5}\sum\left|\boldsymbol{Q}_{\alpha\beta}^{(e)}\right|^2 \tag{S8}$$

$$I_{Q^{(m)}} = \frac{\omega^6}{40c^5}\sum\left|\boldsymbol{Q}_{\alpha\beta}^{(m)}\right|^2 \tag{S9}$$

$$I_T = \frac{2\omega^6}{3c^5}\left|\boldsymbol{T}\right|^2 \tag{S10}$$

Supporting Note S5. Experimental procedure

Figure S4 illustrates the transmissive terahertz time-domain spectroscopy (THz-TDS) system employed in this study. Because terahertz pulses are highly sensitive to polar molecules—particularly atmospheric water vapor—the rotational transitions of water molecules induce intense absorption lines within the terahertz regime. This severely degrades both the signal-to-noise ratio (SNR) and the baseline flatness of the experimental spectra.

To mitigate these effects, strict environmental controls were implemented. The entire optical assembly was enclosed in a sealed acrylic chamber, with the internal humidity monitored in real time using a high-precision digital hygrometer. Prior to each measurement, the chamber was purged with dry nitrogen until the relative humidity (RH) stabilized below 5%.

The experimental procedure was executed as follows:

- **Reference Measurement:** An air reference signal was first acquired with an empty sample holder at the focal position.
- **Sample Measurement:** The sample was then loaded into the holder. Because sample exchange introduces ambient air and inevitably increases internal humidity, the chamber was resealed and re-purged with dry nitrogen before each subsequent measurement.

Data acquisition commenced only after the humidity re-stabilized below the 5% threshold, thereby ensuring high consistency and reproducibility across all datasets. To optimize the

spatial layout on our optical breadboard, a dedicated pellicle beam combiner was utilized to co-linearly direct the probe beam and the THz beam into the detector.

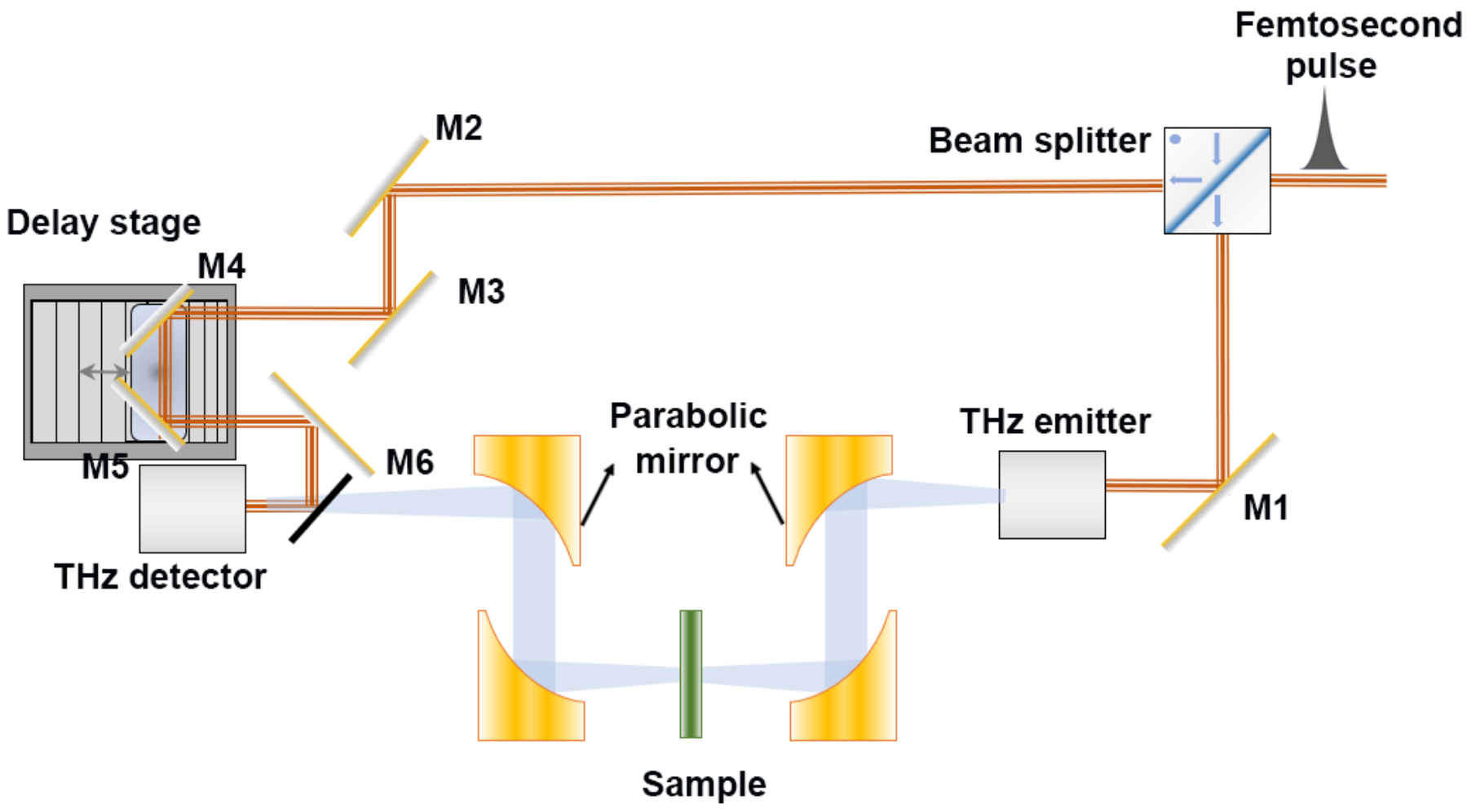

Figure S4. The transmissive terahertz time-domain spectroscopy

Supporting Note S6. Quantitative fitting and parameter characterization of QBIC modes

To quantitatively extract the physical characteristics of QBIC modes, we employed the Fano resonance model to fit the experimental and simulated transmission spectra. The Fano lineshape, originating from the coherent interference between a discrete bound state and a continuous radiative background, accurately captures the asymmetric resonance features characteristic of QBIC modes [1, 2]. The fitting formula is expressed as follows:

$$T(\omega) = T_0 + A \bullet \frac{(W+q)^2}{W^2+1} \frac{1}{(1+q^2)} \tag{S11}$$

where T_0 is background transmittance, representing the non-resonant response far from the resonance frequency. A is Resonance amplitude factor. q is the Fano factor, a dimensionless parameter that characterizes the asymmetry of the lineshape. Its value reflects the ratio of the transition amplitudes between the discrete and continuum states. A large $|q|$ results in a symmetric Lorentzian profile, whereas $q\approx0$ corresponds to an anti-resonance window. W is reduced frequency, which is defined as $W=2(\omega-\omega_0)/\gamma$. ω_0 is resonant center frequency, and γ represents resonant bandwidth.

Upon completing the fitting process, the Q-factor of QBIC mode is determined by the ratio of the extracted center frequency ω_0 to the linewidth γ:

$$Q = \frac{\omega_0}{\gamma} \tag{S12}$$

This method effectively decouples the asymmetric background from the intrinsic resonance width, ensuring that the extracted Q-factors are accurate and reliable even in the presence of significant background noise or loss.

Supporting Note S7. Numerical evaluation of experimental non-idealities on QBIC Q-factor degradation

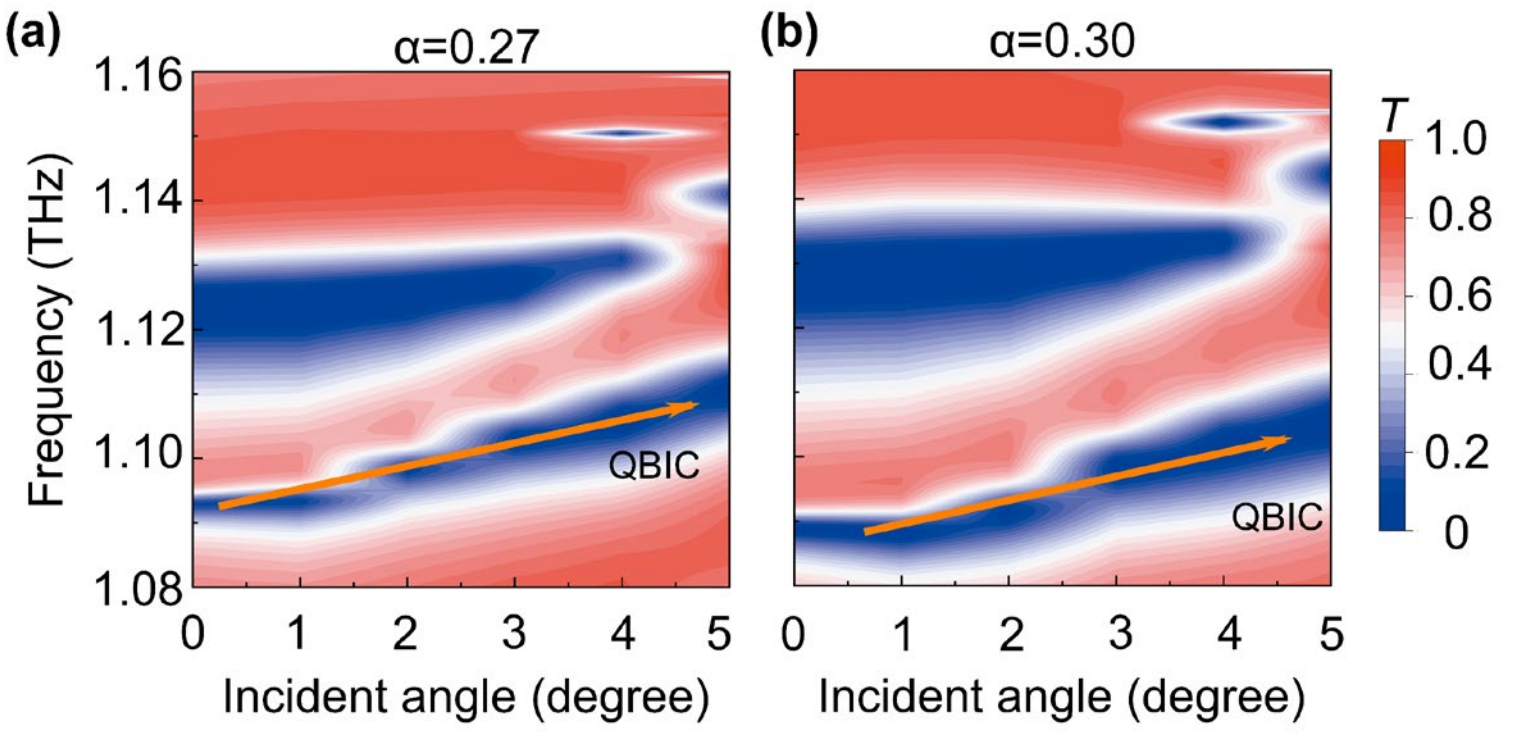

Figure S5 Simulated transmittance maps as a function of incident angle for asymmetry parameters (a) α=0.27 and (b) α=0.30. The color scale represents the transmittance (T) ranging from 0 (blue) to 1 (red). The orange arrows indicate the blueshift of the QBIC resonance frequency with increasing incident angle.

To investigate the effect of THz beam divergence on the experimentally measured transmission spectra, we performed full-wave simulations of the metasurface transmittance under oblique incidence at angles θ ranging from 0° to 5°. In these simulations, the polyimide substrate was modeled with experimentally measured dielectric parameters (ε=3.5, tanδ=0.0027) to reproduce realistic experimental conditions. Figure S5 shows the simulated transmittance maps as a function of incident angle for α=0.27 and α=0.30. At normal incidence (θ=0°), both structures exhibit a well-defined QBIC resonance manifesting as a sharp transmission dip near 1.09 THz. The resonance frequencies and Q-factors extracted by Fano fitting under each incident angle are summarized in Table S1. As the incident angle increases from 0° to 5°, the QBIC resonance undergoes a progressive blueshift (indicated by the orange arrows) accompanied by continuous linewidth broadening and a reduction in Q-factor, reflecting the angle-dependent dispersion of the leaky resonance mode. Specifically, for α=0.27, the Q-factor decreases from 230.17 at θ=0° to 55.34 at θ=5°; for α=0.30, it decreases from 159.56 at θ=0° to 83.03 at θ=5°. Notably, at the same incident angle, the resonance dip for α=0.30 is deeper and broader than that for α=0.27,

consistent with the higher radiative loss associated with the larger asymmetry parameter. It is worth noting that the simulation data exhibit minor non-monotonic fluctuations at certain angles (e.g., between θ=1° and 2° for α=0.27, and between θ=4° and 5° for α=0.30). These fluctuations are considered normal statistical variations stemming from the following factors:

- **Grid Discretization Effects:** In full-wave simulations of oblique incidence, slight perturbations in boundary condition matching and numerical noise induced by the mesh discretization can occur.
- **Fano Fitting Inherent Challenges:** To extract Q_{total}, we employed the Fano resonance model to separate the asymmetric line shape. In these simulations, we incorporated the experimentally measured material losses of the polyimide substrate (complex permittivity ε=3.5 and dielectric loss tangent tanδ=0.0027) to faithfully represent the physical conditions. When oblique incidence leads to non-uniform broadening and blue-shift of the resonance peak, the overlap between the transmission background and the resonant line shape becomes more complex. For such low-Q, broad resonance peaks accompanied by significant background absorption, the Fano fitting process inherently involves minor extraction errors regarding the extremum and linewidth.

Despite minor fitting deviations at individual data points, these do not alter the macroscopic physical trend of continuous QBIC resonance broadening and sharp Q-factor degradation under oblique incidence. This observation provides sufficient justification that the finite divergence angle of the focused terahertz beam in the experiment significantly contributes to the lower measured Q_{total}.

Table S1. QBIC resonance frequencies and total Q-factors (Q_{total}) extracted by Fano fitting at different incident angles for α=0.27 and α=0.30.

		θ=0°	θ=1°	θ=2°	θ=3°	θ=4°	θ=5°
α=0.27	Frequency (THz)	1.093	1.088	1.092	1.098	1.102	1.106
	Q_{total}	230.17	104.78	109.61	75.54	59.80	55.34
α=0.30	Frequency (THz)	1.089	1.094	1.099	1.103	1.106	1.111
	Q_{total}	159.56	138.76	106.60	83.67	73.26	83.03

References

1. A. E. Miroshnichenko, S. Flach, and Y. S. Kivshar, “Fano Resonances in Nanoscale Structures,” *Reviews of Modern Physic* (2010): 2257-2298, https://doi.org/10.1103/RevModPhys.82.2257.
2. M. F. Limonov, “Fano Resonance for Applications,” *Advances in Optics and Photonics* (2021):

703-771, https://doi.org/10.1364/AOP.420731.
